# Phenotypic Variability and Diagnostic Characteristics of Pediatric Osteogenesis Imperfecta: A 10-Year Multicenter Cohort Study from Three Tertiary Pediatric Hospitals in Bucharest, Romania

**DOI:** 10.3390/diagnostics16131997

**Published:** 2026-06-26

**Authors:** Alexandru Dinulescu, Mara-Elena Stăiculescu, Irina Dijmărescu, Mirela-Luminița Pavelescu, Daniela Păcurar, Alexandru Ulici

**Affiliations:** 1 Department of Pediatrics, “Carol Davila” University of Medicine and Pharmacy, 020021 Bucharest, Romania; alexandru.dinulescu@drd.umfcd.ro (A.D.); mara-elena.staiculescu25@rez.umfcd.ro (M.-E.S.); irina.dijmarescu@umfcd.ro (I.D.); 2 Department of Pediatrics, Emergency Hospital for Children “Grigore Alexandrescu”, 011743 Bucharest, Romania; 3 Department of Pediatric Orthopedics, “Carol Davila” University of Medicine and Pharmacy, 020021 Bucharest, Romania; alexandru.ulici@umfcd.ro; 4 Department of Pediatric Orthopedics, Emergency Hospital for Children “Grigore Alexandrescu”, 011743 Bucharest, Romania

**Keywords:** osteogenesis imperfecta, pediatric, Lobstein disease, brittle bone disease, bone fragility, rare disease, Romania, multicenter cohort, Sillence classification

## Abstract

**Background/Objectives**: Osteogenesis imperfecta (OI) is a rare hereditary connective tissue disorder characterized by bone fragility, recurrent fractures, and variable extra-skeletal manifestations. There are scarce epidemiological and clinical data from Eastern Europe, including Romania. This study aimed to characterize the phenotypic spectrum and diagnostic features of pediatric OI in a Romanian multicenter cohort. **Methods**: A retrospective, multicenter observational study was conducted over a 10-year period (January 2014–December 2024) in three tertiary pediatric referral centers in Bucharest, Romania. Children with a diagnosis of OI based on clinical, radiological, and, where available, molecular criteria were included. Clinical, phenotypic, genetic, and therapeutic data were extracted from medical records. Statistical analyses included the Mann–Whitney U test, Kruskal–Wallis test with Bonferroni correction, Fisher’s exact test, Kaplan–Meier survival analysis, and Spearman correlation. **Results**: Forty-seven patients were included (53.2% female; median age at diagnosis 36 months, IQR 5–87). OI type I was the most frequent subtype (42.6%), followed by type III (29.8%) and type IV (21.3%). Molecular genetic testing was performed in 40.4% of patients; among genetically tested patients, COL1A1 variants represented the most common finding (52.6%). The median number of documented fractures was 5 (IQR 3–9), with a significantly higher annual fracture rate in type III compared to type I (0.99 vs. 0.34 fractures/year, *p* = 0.019). Short stature was the most frequent skeletal manifestation (66%), with significantly more severe growth impairment in type III compared to type I (−4.38 ± 1.67 vs. −1.56 ± 1.03 SD, *p* < 0.001). Blue sclerae was present in 87.2% of patients and dentinogenesis imperfecta in 68.1%. Cryptorchidism was identified in 50% of male patients. Developmental motor milestones were significantly delayed in type III OI patients, with 10.6% failing to achieve independent walking by last follow-up. A strong positive correlation was observed between age at first fracture and age at diagnosis (Spearman R = 0.764, *p* < 0.001), with type I patients diagnosed significantly later than type III (median 71.5 vs. 6.5 months, *p* = 0.006). **Conclusions**: This study provides the most comprehensive phenotypic characterization of pediatric OI reported from Romania to date. Our findings confirm established genotype–phenotype correlations and underscore the diagnostic challenge of milder OI forms. The high prevalence of cryptorchidism in male patients represents a clinically relevant finding needing prospective validation. The data highlight the need for expanded molecular diagnostic capacity, increased disease awareness among frontline clinicians, and the development of a national OI registry to support longitudinal research and integration with European rare bone disease networks.

## 1. Introduction

Osteogenesis imperfecta (OI), also known as brittle bone disease or Lobstein disease, is a rare inherited connective tissue disorder characterized by increased bone fragility, recurrent fractures, variable skeletal deformities and highly variable secondary connective tissue findings, including blue sclerae, dentinogenesis imperfecta, hearing loss, and joint hypermobility [1,2,3,4,5,6]. The condition is primarily caused by mutations affecting type I collagen synthesis, leading to impaired bone strength and structural integrity. The majority of affected individuals carry structural or quantitative defects in the COL1A1 and COL1A2 genes, encoding the α1(I) and α2(I) chains of type I collagen, with autosomal dominant (AD) inheritance predominating, but autosomal recessive (AR) and X-linked variants of the disease have also been described [7,8,9]. The latest advances in molecular genetics have broadened the OI classification to 22 recognized types, with newly identified variants affecting pathways beyond collagen synthesis, including osteoblast differentiation and bone mineralization [5,10,11,12].

The clinical spectrum of OI is very broad. Phenotypic variability ranges from prenatal fractures with lethal outcomes to mild forms with few fractures and normal stature and even individuals that carry identical mutations may have different disease severity, underscoring the contribution of modifier genes and environmental factors [13,14,15]. The most widely used clinical classification is the Sillence classification, proposed in 1979, which categorizes OI into 4 syndromic groups based on clinical features and mode of inheritance. This classification distinguished type I (mild, non-deforming phenotype), type II (perinatally lethal), type III (severe, non-lethal, progressively deforming), and type IV (moderate severity, intermediate between types I and III) [16,17]. Although advances in molecular genetics have expanded the classification, the Sillence system remains widely used in clinical practice [18,19,20].

OI is considered a rare disease, with an estimated prevalence of approximately 1 in 10,000 to 1 in 20,000 live births worldwide [1,21,22]. However, OI is considered a significantly underdiagnosed disease, and its true prevalence has not been systematically explored across many regions of the world [21,23]. Population-based registry data providing robust epidemiological estimates and genotype–phenotype insights remain insufficient, with most available evidence derived from registry-based or single-center studies conducted in a limited number of high-income countries, predominantly in Northern and Western Europe, North America, and East Asia [24,25,26,27,28]. Data from Eastern European populations remain scarce and largely limited to small single-center studies or genetic analyses focused on mutational spectra, as reported from Ukraine, Estonia and Poland [29,30,31]. In Romania, published data are restricted to a recent case series from a regional genetics center located in Craiova, Dolj [21].

Comprehensive multicenter pediatric cohort studies describing the full clinical and phenotypic spectrum of OI in this region are lacking. Given the limited data available from Romanian centers, this study aimed to characterize the phenotypic spectrum, extra-skeletal manifestations, and diagnostic features of pediatric OI in a 10-year multicenter cohort from 3 tertiary hospitals in Bucharest, Romania.

## 2. Materials and Methods

### 2.1. Study Design and Setting

This was a retrospective, multicenter observational study conducted over a 10-year period (1 January 2014–31 December 2024) in 3 tertiary pediatric referral centers: “Grigore Alexandrescu” Emergency Clinical Hospital for Children, “Victor Gomoiu” Clinical Hospital for Children and “Marie Curie” Emergency Clinical Hospital for Children, located in Bucharest, Romania. These institutions are major tertiary referral centers for orthopedic diseases and among the largest pediatric hospitals in the country, providing care for children with rare and complex conditions, including osteogenesis imperfecta.

### 2.2. Study Population

**Figure 1:**
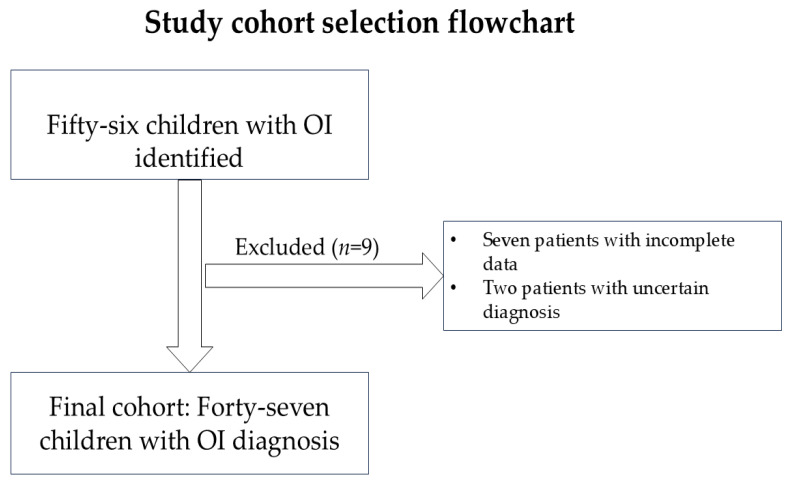
Study cohort selection.

We included pediatric patients (aged < 18 years) diagnosed with osteogenesis imperfecta during the study period. Diagnosis was established based on clinical and radiological findings, with genetic confirmation where available. We found 56 patients in the electronic registers of the 3 hospitals. Patients with incomplete medical records or uncertain diagnosis were excluded (9 patients); the remaining 47 patients were included in this study (Figure 1).

### 2.3. Data Collection

Data were retrospectively extracted from electronic and paper medical records using a standardized data collection form.

The following variables were collected:

- Demographic data: age at diagnosis, sex, area of provenance, year of diagnosis;
- Clinical presentation: mode of presentation at diagnosis (e.g., fracture, bone deformity, incidental diagnosis);
- Phenotypic classification: Sillence type;
- Skeletal manifestations: total number of fractures, fracture localization, presence of bone deformities;
- Extra-skeletal manifestations: blue sclera, dentinogenesis imperfecta, hernias, and other associated features;
- Family history: presence of affected relatives;
- Genetic testing, where it was available. (Details regarding sequencing platforms were not consistently available because testing had been performed in multiple external laboratories over the study period.) Access to molecular diagnostics was limited because genetic testing for osteogenesis imperfecta was not reimbursed by the Romanian National Health Insurance System during the study period, and the associated costs had to be borne entirely by patients’ families;
- Therapeutic data: initiation of bisphosphonate therapy and presence of intramedullary rodding;
- Complications.

### 2.4. Definitions

Symptom prompting medical evaluation was defined as the first reported clinical symptom that led to medical assessment and diagnostic work-up for osteogenesis imperfecta. Clinical diagnosis of OI was established by pediatricians and orthopedic surgeons based on the combination of recurrent low-energy fractures, skeletal deformities, characteristic extra-skeletal manifestations (e.g., blue sclerae, dentinogenesis imperfecta), family history, and supportive radiographic findings.

The annual fracture rate was calculated as the number of documented fractures divided by the duration of the observation period in years.

Short stature was defined as a height-for-age Z-score below −2 standard deviations according to age- and sex-adjusted pediatric growth references [32].

Antenatal diagnosis was established following routine prenatal ultrasonography demonstrating skeletal abnormalities suggestive of osteogenesis imperfecta, including long-bone bowing and/or fractures, in the context of a positive family history of osteogenesis imperfecta.

Age at diagnosis was defined as the age at which osteogenesis imperfecta was first formally documented in the medical record by the treating physician.

### 2.5. Statistical Analysis

Statistical analysis was performed using IBM SPSS Statistics version 25 (IBM Corp., Armonk, NY, USA). Quantitative variables were tested for normal distribution using the Shapiro–Wilk test, normally distributed variables were expressed as mean ± standard deviation (SD), while non-normally distributed variables were reported as median and interquartile range (IQR). Quantitative variables were tested between independent groups using the Mann–Whitney U test and the Kruskal–Wallis test. Comparisons between two independent groups were performed using the independent samples *t*-test (Welch correction was applied when the assumption of homogeneity of variances was violated).

Fisher’s exact test was used to determine the nonrandom associations between categorical variables, with the Bonferroni method used for correction. Time to attainment of independent sitting and independent walking was analyzed using Kaplan–Meier survival analysis. Children who had not achieved independent sitting/walking at the last follow-up were treated as censored observations. Group comparisons were performed using the log-rank test. Correlations between continuous variables with non-normal distribution were assessed using Spearman’s rank correlation coefficient. A *p*-value < 0.05 was considered statistically significant.

## 3. Results

### 3.1. Study Population Main Characteristics

We included 47 pediatric patients with an OI diagnosis in the study.

The follow-up time followed a normal distribution (*p* = 0.780) with an average of 81.36 ± 38.37 months. Female patients represented 53.2% of the cohort (*n* = 25), while 46.8% were male (*n* = 22). Most patients originated from urban areas (55.3%).

A positive family history of osteogenesis imperfecta was identified in 31.9% of cases. Regarding disease severity according to the Sillence classification, type I was the most frequent form, accounting for 42.6% of patients, followed by type III (29.8%) and type IV (21.3%). Less common forms included type V (4.3%) and type XVII (2.1%). Genetic testing had not been performed in the majority of patients (59.6%). Among patients who underwent molecular testing, the most frequently identified pathogenic variants involved the COL1A1 gene (21.3%), followed by COL1A2 mutations (8.5%). One patient carried an IFITM5 mutation and one patient a SPARC mutation. In three patients, genetic testing was performed but no pathogenic variant was identified. Of the two patients classified as having OI type V, one had molecular confirmation of an IFITM5 variant, while the second diagnosis was established on the basis of typical radiographic findings, including hyperplastic callus formation and ossification of the interosseous membrane.

Most patients received intravenous bisphosphonate therapy. Pamidronate was administered in 74.5% of cases, while 6.4% received zoledronic acid. Nine patients (19.1%) had not received bisphosphonate treatment at the time of analysis. Orthopedic surgical intervention using the Sofield–Millar procedure was performed in 48.9% of patients. Most of them, 34 (72.3%), had undergone physiotherapy.

Epilepsy was identified in 2 patients (4.3%). Ehlers–Danlos overlap phenotype was identified in 5 patients (10.6%), 4 of whom had COL1-related OI; in 1 patient, genetic testing was not performed.

Detailed demographic and clinical characteristics of the cohort are summarized in Table 1.

Fracture was the most common symptom prompting medical evaluation at diagnosis, accounting for 63.8% of cases (*n* = 30). Skeletal deformities represented the second most frequent presenting feature, observed in 21.3% of patients (*n* = 10). Antenatal diagnosis and blue sclera accounted for 4.3% of cases (*n* = 2) each. Less frequent presenting manifestations included inguinal hernia (2.1%), hypotonia (2.1%), and gait disturbances (2.1%) (Table 2).

### 3.2. Fractures

The vast majority, 45 patients (95.7%), had at least one fracture. Two patients had no documented fractures at the time of data collection. One was diagnosed antenatally because of characteristic ultrasonographic findings and family history, while the second fulfilled clinical diagnostic criteria based on extra-skeletal manifestations and a positive family history. The number of documented fractures had a non-normal distribution (*p* < 0.001) with a median of 5 (3–9) fractures. In those with at least one fracture, the age of the first fracture had a non-normal distribution (*p* < 0.001) with a median of 27 (0.5–71) months. All 45 of them (100%) had at least one long bone fracture. Two of them (4.4%) had spine or rib fractures, and one of them (2.2%) had a pelvic fracture. Almost all of them, 42 (93.3%), had at least one long bone fracture in the lower limb, and 32 (71.1%) had at least one fracture in the upper limb.

**Figure 2:**
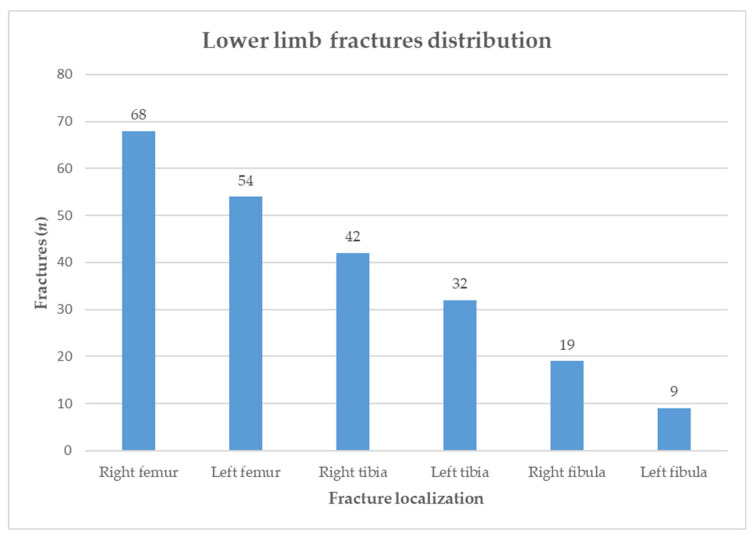
Lower limb fractures distribution.

In total, there were 224 fractures at the lower limb level, with most of them, 122 (54.4%), being femur fractures, followed by tibia, 33.1% (*n* = 74%), and fibula, 12.5% (*n* = 28) (Figure 2).

**Figure 3:**
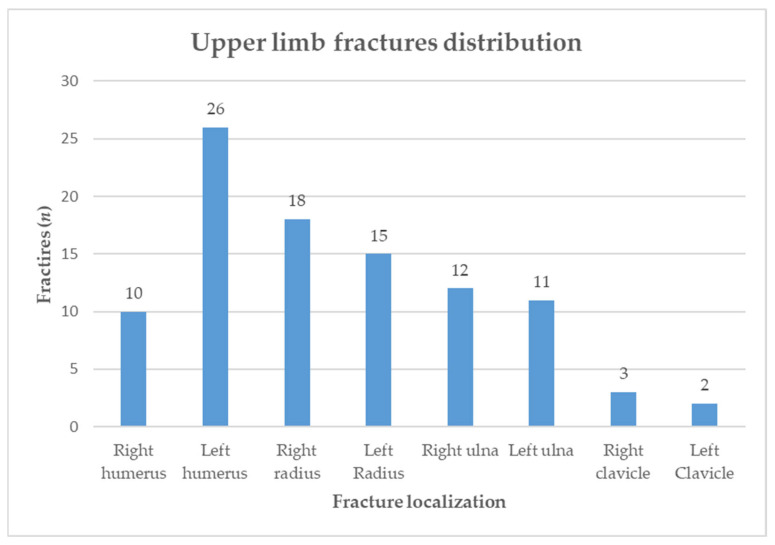
Upper limb fractures distribution.

There were 97 fractures at the upper limb level, with most of them, 37.1% (*n* = 36), being humerus fractures, followed by radius, 34% (*n* = 33), ulna, 23.7% (*n* = 23), and clavicles, 5.2% (*n* = 5) (Figure 3).

The annual fracture rate had a non-normal distribution (*p* < 0.001) with a median of 0.45 (0.28–1) fractures/year. There was no difference in annual fracture rate by sex (*p* = 0.654).

The annual fracture rate differed significantly across Sillence types (*p* = 0.019).

Post hoc pairwise analysis with Bonferroni correction demonstrated a significantly higher fracture rate in patients with Sillence type III compared to type I (adjusted *p* = 0.019).

**Figure 4:**
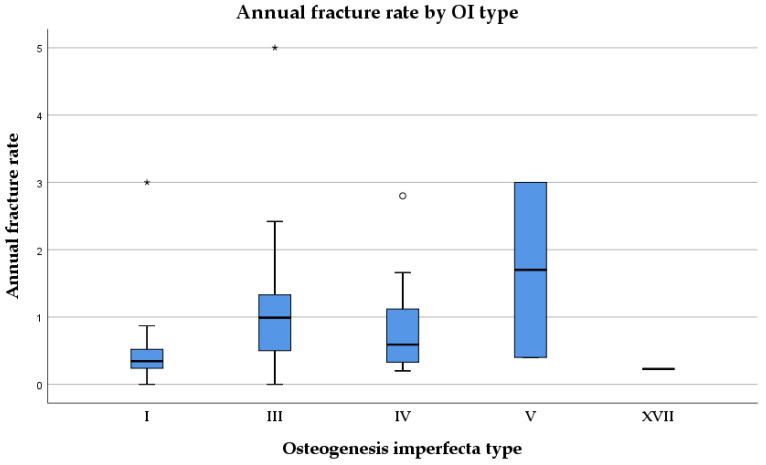
Annual fracture rate by OI type. The interior bars indicate the medians while the whiskers extend to the maximum and minimum of the data; ◦ = outlier; * = far outlier.

Patients with type III OI had the highest median annual fracture rate, 0.99 (0.48–1.56) fractures/year, whereas patients with type I presented substantially lower fracture rates, 0.34 (0.23–0.53) fractures/year (Figure 4).

We found a higher annual fracture rate in those who received bisphosphonate therapy (0.5 fractures/year) than those who did not (0.37 fractures/year), but no significant difference in annual fracture rate was observed (*p* = 0.357).

### 3.3. Skeletal Manifestations

**Figure 5:**
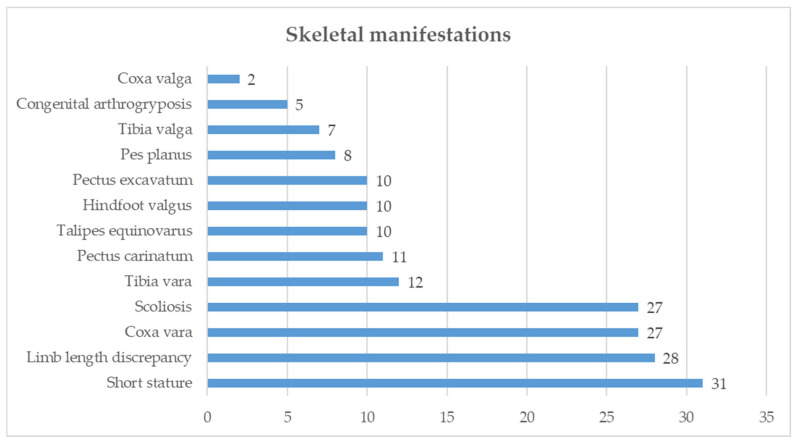
Distribution of skeletal manifestations.

All of them had at least one skeletal manifestation. The most frequent one was short stature, found in 31 patients (66%), followed by limb length discrepancy in 28 patients (59.6%), and both coxa vara and scoliosis in 27 (57.4%) patients (Figure 5).

The Height Z score had a normal distribution (*p* = 0.337) with an average of −2.62 ± 1.8 SD. A significant difference in height Z-score was observed between type I and type III groups (Welch *t*-test, *p* < 0.001), with more severe growth impairment in type III (−1.56 ± 1.03 vs. −4.38 ± 1.67). Other pairwise comparisons did not reach statistical significance (*p* > 0.05).

No significant association was observed between OI type and scoliosis (*p* = 0.295).

Limb length discrepancy was identified in all patients with Sillence type III and type V OI, compared to only 30% of patients with type I OI (*p* < 0.001). Similarly, coxa vara was more commonly observed in type III, affecting 85.7% of patients with type III OI, whereas only 30% of patients with type I OI presented this deformity (*p* = 0.009) (Table 3).

### 3.4. Extra-Skeletal Manifestations

**Figure 6:**
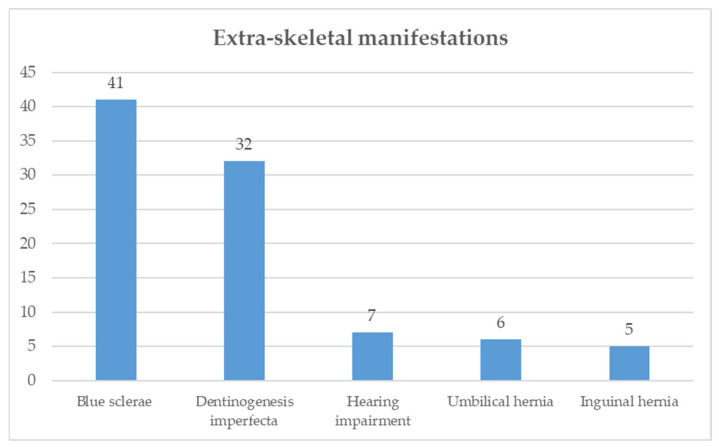
Distribution of extra-skeletal manifestations.

In only one subject (2.1%), an extra-skeletal manifestation was not identified. The most common reported one was blue sclera at 87.2% (*n* = 41), followed by dentinogenesis imperfecta reported at 68.1% (*n* = 32) (Figure 6).

Cryptorchidism history was found in 50% (*n* = 11) of male patients (*n* = 22). There was no association between cryptorchidism and OI type (*p* = 0.054).

Dentinogenesis imperfecta and hearing impairment were not associated with any OI type (*p* = 0.555; *p* = 0.455).

Blue sclera was associated more frequently with OI type I and III than with OI type IV (100% vs. 60%) (*p* < 0.001).

### 3.5. Developmental Motor Milestones

#### 3.5.1. Head Control

**Figure 7:**
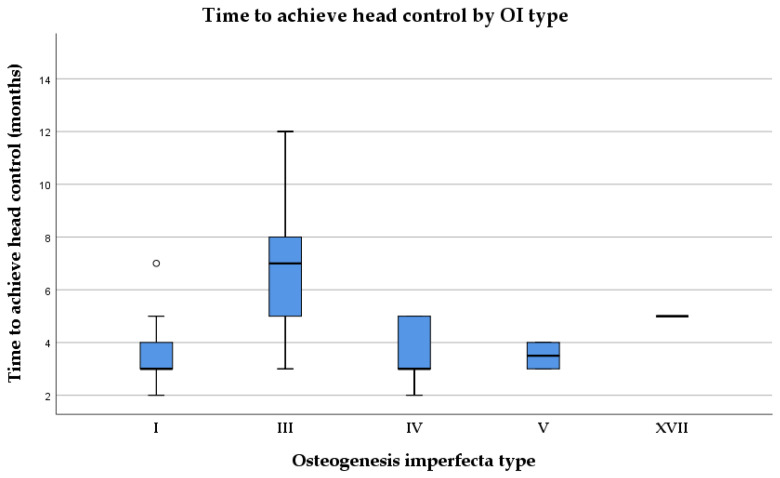
Time to achieve head control by OI type. The interior bars indicate the medians while the whiskers extend to the maximum and minimum of the data; ◦ = outlier.

All of the patients included in the study achieved head control. The time of achievement had a non-normal distribution (*p* < 0.001) with a median of 4 (3–5) months. Post hoc pairwise analysis with Bonferroni correction demonstrated a significantly longer time to achieve head control in patients with Sillence type III compared to type I and type IV (adjusted *p* < 0.001). Patients with type III OI had the longest time to achieve head control, 7 (4.75–8.25) months, whereas patients with type I and type IV presented substantially less time for this achievement, 3 (3–4) months for type I and 3 (2.75–5) months for type IV (Figure 7).

#### 3.5.2. Sitting Without Support

**Figure 8:**
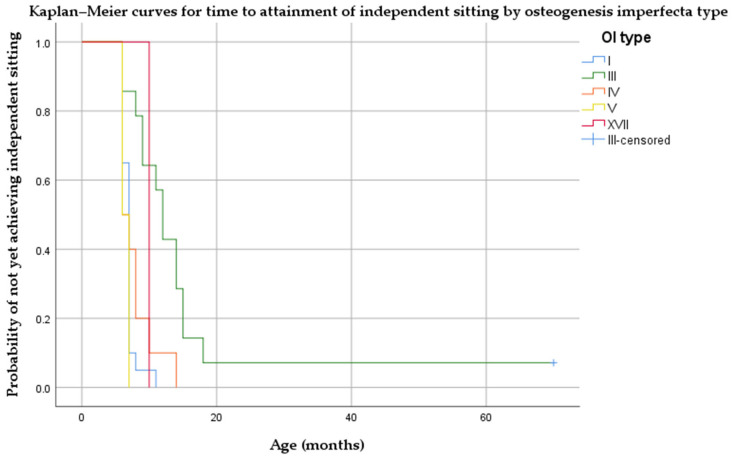
Kaplan–Meier curves for time to independent sitting according to OI type.

Only one patient (2.1%) at the time of this study did not achieve sitting without support; it was a 70-month-old type III OI patient. For the remaining 46 patients who achieved this milestone, the age at achievement had a non-normal distribution with a median of 7 (6–10) months. Time to independent sitting differed significantly between OI types (log-rank test: χ^2^ = 21.165, *p* < 0.001). Children with OI type III exhibited a delayed acquisition of independent sitting compared with other groups, with a higher estimated median age at milestone attainment (median: 12.0 months; IQR: 10.2–13.8). In contrast, earlier achievement was observed in OI type I (median: 7.0 months; IQR: 6.8–7.2), type IV (median: 6.0 months), and type V (median: 6.0 months) (Figure 8). One case in the OI type III group was censored due to failure to achieve independent sitting by the last follow-up.

#### 3.5.3. Independent Walking

**Figure 9:**
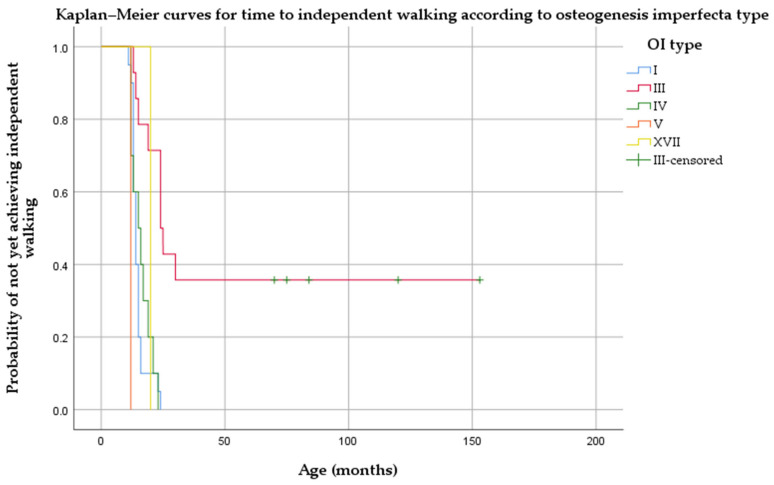
Kaplan–Meier curves for time to independent walking according to OI type.

Five patients (10.6%) did not achieve independent walking at the time of this study. Their age at the last follow-up ranged from 75 to 153 months, and all were classified as having OI type III. Time to independent walking differed significantly between OI types (χ^2^ = 31.973, df = 4, *p* < 0.001). Children with OI type III exhibited a delayed acquisition of independent walking compared with other groups, with a higher estimated median age at milestone attainment of 24 (19–24) months. In contrast, earlier achievement was observed in type I (median: 14.0 months), type IV (median: 15.0 months), and type V (median: 12.0 months) (Figure 9). Five censored cases were observed in the type III group, reflecting children who had not achieved independent walking at the last follow-up.

#### 3.5.4. Gait Disturbances

Gait disturbances were present in over half of them (53.2%). There was an association between OI type and the presence of gait disturbances, with them being reported more frequently in type III (92.9%) than type I (25%) (*p* < 0.001).

### 3.6. Complications

The most common complication was pseudarthrosis (34.1%), followed by postoperative abscess (8.5%) and deep vein thrombosis and bursitis (2.1% each). Type III was more frequently associated with the presence of complications than type I, 92.9% vs. 45% (*p* = 0.22).

There was no association between the presence of pseudarthrosis and the OI type (*p* = 0.504).

### 3.7. Age at Diagnosis

Age at diagnosis had a non-normal distribution (*p* < 0.001) and a median of 36 (5–87) months. There was no association between age at diagnosis and sex (*p* = 0.623), area of provenance (*p* = 0.715), symptom prompting medical evaluation (*p* = 0.272) or a known relative with OI (*p* = 0.137).

A strong positive correlation between age at first fracture and age at diagnosis (R = 0.764, *p* < 0.001) was identified.

**Figure 10:**
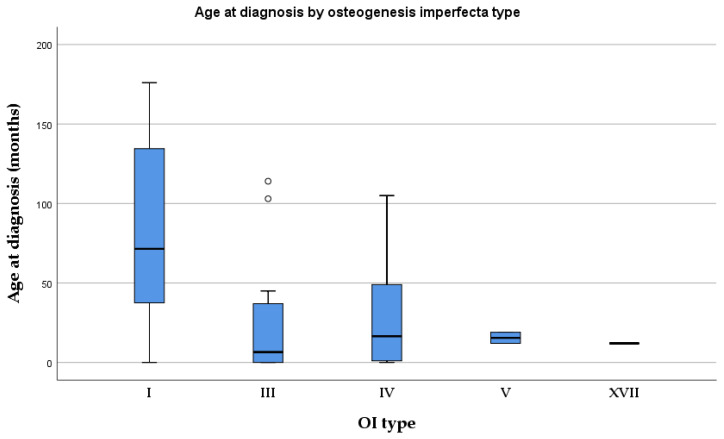
Age at diagnosis by OI type. The interior bars indicate the medians while the whiskers extend to the maximum and minimum of the data; ◦ = outlier.

Post hoc pairwise analysis with Bonferroni correction demonstrated a significantly earlier diagnosis age in patients with type III compared to type I (adjusted *p* = 0.006). Patients with type III OI had a median age at diagnosis of 6.5 (0–39) months, whereas patients with type I had a median age at diagnosis of 71.5 (36.75–138.25) months (Figure 10).

## 4. Discussion

This multicenter cohort study provides a comprehensive description of the phenotypic spectrum and diagnostic characteristics of pediatric osteogenesis imperfecta in the Bucharest region, Romania, representing the most extensive pediatric OI dataset reported from this country to date. Our findings are consistent with previously published European and international cohort studies, while also reflecting features specific to our regional healthcare context.

The present study was not population-based; therefore, incidence estimates could not be reliably calculated. To our knowledge, no nationwide epidemiological studies of pediatric osteogenesis imperfecta have been conducted in Romania to date. Internationally, OI has been estimated to occur in approximately 1 in 10,000–20,000 live births [1]. Recently, a nationwide pediatric registry study from Türkiye by Görgün et al. (2024) reported a prevalence of 11.6 per 100,000 children and a median annual incidence of 31.5 per 100,000 live births between 2016 and 2022, highlighting regional differences in case ascertainment and registry-based methodologies [33].

Although 31.9% of patients reported a positive family history of OI, this proportion should not be interpreted as representing the true frequency of de novo disease. The limited availability of molecular testing and incomplete family investigations may have led to underestimation of inherited forms, with previous studies reporting autosomal dominant inheritance in approximately 85–90% of OI cases [34].

### 4.1. Disease Distribution and Genetic Findings

The predominance of OI type I in our cohort (42.6%) aligns with the distribution reported in large European registries and multicenter studies, where type I is identified as the most frequent form, typically accounting for 40–60% of OI cases [34,35,36]. In our cohort, type III and type IV have higher rates (29.8% and 21.3%) than the ones described in the literature, but this may reflect referral patterns to tertiary centers, as more severe phenotypes are disproportionately represented in specialized hospital settings [3,37].

Among genetically tested patients, COL1A1 variants were the most common (52.6%), followed by COL1A2 (21%), consistent with the well-established predominance of type I collagen gene mutations in OI populations worldwide [29,38,39]. The identification of one IFITM5 mutation (associated with OI type V) and one SPARC mutation (associated with OI type XVII) confirms the presence of non-collagen OI subtypes in the Romanian pediatric population, though their frequency remains low, as expected given the rarity of these forms [40,41,42,43].

### 4.2. Fracture Burden and Annual Fracture Rate

The median number of documented fractures in our cohort was 5 (IQR 3–9), with a median annual fracture rate of 0.45 fractures/year. The significantly higher fracture rate in OI type III compared to type I (0.99 vs. 0.34 fractures/year, *p* = 0.019) is consistent with the known severity gradient across Sillence types and is in concordance with findings from comparable pediatric cohorts [44,45,46,47]. Long bone fractures predominated, with femur fractures representing the most frequent lower limb injury (54.4%) and humerus fractures the most common upper limb injury (37.1%), a distribution that is consistent with previous reports [47,48].

Even though it was not statistically significant (*p* = 0.357), the ones that received bisphosphonate therapy had a higher annual fracture rate (0.5 vs. 0.37 fractures/year), but this finding should be interpreted with caution. The apparently higher fracture burden observed among patients receiving bisphosphonates likely reflects treatment allocation bias, whereby patients with more severe phenotypes and higher baseline fracture rates were preferentially selected for therapy. Therefore, comparisons between treated and untreated patients may not accurately reflect treatment efficacy in this retrospective cohort. Because pre- and post-treatment fracture rates were not analyzed separately in this study, no conclusions regarding treatment efficacy can be drawn.

### 4.3. Skeletal Deformities

Short stature was the most frequent skeletal manifestation, affecting 66% of patients, with a mean height Z-score of −2.62 ± 1.80 SD across the cohort, a finding well known in the literature [49,50]. The significantly more severe growth impairment in OI type III compared to type I (−4.38 ± 1.67 vs. −1.56 ± 1.03, *p* < 0.001) reflects the progressive bone deformity and vertebral involvement characteristic of this subtype [51]. These findings are in keeping with published growth data for pediatric OI, in which patients with type III consistently demonstrate the most pronounced height deficits [26,52].

Limb length discrepancy and coxa vara were significantly more prevalent in type III and type V patients (100% and 85.7% for type III, respectively), compared to type I patients (30% for both deformities, *p* < 0.001 and *p* = 0.009). This high prevalence in severe OI subtypes may reflect the cumulative effect of repeated proximal femoral fractures, abnormal bone modeling, and inadequate mechanical loading, a pattern well-recognized in the OI literature [53,54,55,56,57].

### 4.4. Extra-Skeletal Manifestations

Blue sclerae were the most prevalent extra-skeletal finding (87.2%), followed by dentinogenesis imperfecta (68.1%).

The high prevalence of blue sclerae, particularly its near-universal presence in types I and III (100%) compared to type IV (60%, *p* < 0.001), is consistent with established genotype–phenotype correlations [58]. Blue sclerae in OI result from the reduced thickness and altered optical properties of the scleral collagen matrix, a consequence of type I collagen structural defects [58,59]. The lower prevalence in type IV reflects the broader phenotypic heterogeneity of this subtype, in which structural collagen mutations may spare scleral involvement [1,58,59].

The prevalence of dentinogenesis imperfecta (68.1%) in our cohort is higher than that reported in some Western European series, where rates of 30–50% are more commonly described [60,61]. This discrepancy may be attributable to ascertainment differences, inclusion of all clinical grades of dentinogenesis imperfecta, or differences in the genetics of the study population. The absence of a statistically significant association between dentinogenesis imperfecta and OI type (*p* = 0.555) has been previously reported and reflects the variable penetrance of this manifestation even within identical mutation classes [60,61,62,63,64].

A particularly noteworthy finding in our cohort is the high prevalence of cryptorchidism, documented in 50% of male patients (11 of 22). This rate substantially exceeds the general population prevalence of 2–4% at birth [65,66]. While the association between OI and cryptorchidism has been sporadically reported in the literature, its systematic documentation in pediatric OI cohorts remains limited [67]. The biological plausibility of this association can be attributed to the role of type I collagen in the structural integrity of the processus vaginalis and gubernaculum, whose normal development is required for testicular descent [68,69]. This finding warrants prospective validation in larger cohorts and may have practical implications for the urological surveillance of male patients with OI.

### 4.5. Developmental Motor Milestones

The analysis of developmental motor milestones revealed significant delays in OI type III patients across all assessed achievements, including head control, independent sitting, and independent walking. Children with type III OI achieved independent sitting at a median of 12 months and independent walking at a median of 24 months, compared to 7 and 14 months, respectively, in type I patients (*p* < 0.001 for both). Notably, five type III patients (10.6% of the total cohort) had not achieved independent walking by the last follow-up, with ages ranging from 75 to 153 months. These findings are consistent with published reports of delayed motor development in severe OI, in which the combination of skeletal fragility, limb deformities, muscular hypotonia, and pain imposes significant constraints on motor acquisition [70,71,72,73,74,75].

### 4.6. Diagnostic Delay

The strong positive correlation between age at first fracture and age at diagnosis (R = 0.764, *p* < 0.001) can suggest that patients who experienced their first fracture at older ages also tended to receive the diagnosis of osteogenesis imperfecta later in life. This finding, combined with the significantly later diagnosis in type I compared to type III patients (median 71.5 vs. 6.5 months, *p* = 0.006), highlights the challenge of timely OI recognition in milder disease presentations. Patients with type I OI, who often lack overt skeletal deformity and may present with only isolated fractures, are particularly susceptible to diagnostic delay and to misattribution of fractures to alternative causes, including non-accidental injury [13,76,77]. This diagnostic vulnerability has important clinical and medicolegal implications and underscores the need for heightened awareness of OI among pediatricians, emergency physicians, and orthopedic surgeons encountering children with unexplained or recurrent fractures.

### 4.7. Limitations

This study has several limitations. The relatively small sample size reflects the inherent rarity of osteogenesis imperfecta in the pediatric population rather than a methodological shortcoming. Despite a 10-year study period encompassing three major tertiary pediatric referral centers in Bucharest, the number of eligible patients remained limited, which is consistent with the reported prevalence of the condition and with cohort sizes described in comparable studies from other countries. This limits the statistical power available for subgroup analyses and limits the generalizability of the findings to the broader Romanian pediatric population.

The retrospective design of the study has several limitations, including potential inconsistencies in documentation across centers and time periods, variability in the completeness of clinical records, and the absence of standardized assessment protocols applied prospectively. Molecular genetic testing was not available for all patients.

The study was conducted exclusively in tertiary urban referral centers in the capital, and the cohort may not be fully representative of the entire Romanian pediatric OI population, with cases managed at regional- or community-level hospitals that may not have been captured. A national registry-based approach would be required to address this limitation in future research.

Skeletal deformities were identified from orthopedic evaluations and radiological reports documented in the medical records. Quantitative measurements such as Cobb angle, neck-shaft angle, and exact limb-length discrepancy were not consistently available and therefore could not be analyzed retrospectively.

Molecular testing was not systematically performed because it was not covered by the national health insurance system, thereby limiting access for many families. Furthermore, detailed molecular reports were not consistently retrievable, and in several cases, only the affected gene had been documented in electronic discharge summaries.

### 4.8. Future Directions

The findings of the present study underscore the need for coordinated, sustained research efforts to improve the understanding and management of OI in the Romanian pediatric population. Several priorities emerge from this work. The establishment of a national OI registry, built through the systematic centralization of clinical, radiological, and molecular data from all pediatric centers across Romania, would represent a critical step forward. Such a registry would enable robust epidemiological estimates, longitudinal follow-up, and meaningful genotype–phenotype analyses at a national level, while also facilitating integration with existing European rare bone disease networks, such as the ERN BOND (European Reference Network for Rare Bone Disorders) [78].

Targeted efforts to improve disease awareness among healthcare professionals are urgently needed. Osteogenesis imperfecta remains underrecognized in clinical practice, particularly in its milder forms, where the absence of overt skeletal deformity may delay or prevent timely diagnosis [79]. Educational initiatives directed at general physicians, pediatricians, emergency medicine specialists, and orthopedic surgeons who are frequently the first point of contact for children presenting with unexplained fractures are essential to reduce diagnostic delays and minimize the risk of missed or misattributed cases. Structured training programs, clinical decision support tools, and the dissemination of diagnostic criteria through national pediatric and orthopedic societies could meaningfully contribute to earlier disease recognition across all regions of the country, including those with more limited access to specialized care.

The development of dedicated multidisciplinary care centers for OI patients is strongly warranted. Optimal management of this condition extends well beyond fracture prevention and cannot be adequately addressed within a single-specialty framework. A comprehensive multidisciplinary team should include a pediatrician with expertise in metabolic bone disease, an orthopedic surgeon, an endocrinologist, a clinical geneticist, a physiotherapist, and a dental specialist for the management of dentinogenesis imperfecta. Equally important, and frequently overlooked in current practice, is the provision of dedicated psychological support and counseling for both patients and their families, given the substantial psychosocial burden associated with a chronic, physically limiting, and often visually apparent condition diagnosed in childhood. Standardized care pathways within such centers would not only improve clinical outcomes but also reduce diagnostic delays and ensure equitable access to evidence-based treatment across different regions of the country. Future multicenter studies, ideally prospective in design and anchored to a national registry infrastructure, will be essential to validate the findings reported here and to inform national guidelines for the diagnosis and management of pediatric OI in Romania.

## 5. Conclusions

This multicenter cohort study provides the most comprehensive phenotypic characterization of pediatric OI reported from Romania to date, confirming established genotype–phenotype correlations across Sillence subtypes and documenting the significant burden of skeletal deformities, extra-skeletal manifestations, and motor developmental delay in this population. The strong correlation between age at first fracture and age at diagnosis underscores the persistent challenge of timely recognition in milder OI phenotypes. The unexpectedly high prevalence of cryptorchidism in male patients (50%) represents a potentially new extra-skeletal association warranting prospective validation in larger cohorts. The significant delays in motor milestone acquisition in type III patients, with 10.6% failing to achieve independent walking by last follow-up, reinforce the need for early, multidisciplinary rehabilitation within structured care pathways. These findings highlight the need for increased disease awareness among frontline clinicians, expanded molecular diagnostic capacity, and the establishment of a national OI registry to support longitudinal research and integration with European rare bone disease networks.

## Figures and Tables

**Table 1:** Descriptive statistics of the population’s main characteristics.

Variable	*N* (%)	Median (IQR)/Average ± SD
**Year of diagnosis**		
2014	4 (8.5%)	
2015	7 (14.9%)	
2016	8 (17%)	
2017	4 (8.5%)	
2018	8 (17%)	
2019	3 (6.4%)	
2020	4 (8.5%)	
2021	3 (6.4%)	
2022	1 (2.1%)	
2023	4 (8.5%)	
2024	0 (0%)	
2025	1 (2.1%)	
**Sex**		
Male	22 (46.8%)	
Female	25 (53.2%)	
	**Age at diagnosis (months)**	36 (5–87)
**Area of provenance**		
Urban	26 (55.3%)	
Rural	21 (44.7%)	
	**Known relative with OI**	
Yes	15 (31.9%)	
No	32 (68.1%)	
	**OI type**	
I	20 (42.6%)	
III	14 (29.8%)	
IV	10 (21.3%)	
V	2 (4.3%)	
XVII	1 (2.1%)	
	**Genetic test**	
Genetic testing not performed	28 (59.6%)	
Genetic testing performed, no pathogenic variant identified	3 (6.4%)	
COL1A1	10 (21.3%)	
COL1A2	4 (8.5%)	
IFITM5	1 (2.1%)	
SPARC	1 (2.1%)	
	**Bisphosphonate therapy**	
No	9 (19.1%)	
Pamidronate	36 (74.5%)	
Zoledronic acid	3 (6.4%)	
	**Sofield-Millar procedure**	
Yes	23 (48.9%)	
No	24 (51.1%)	
	**Physiotherapy**	
Yes	34 (72.3%)	
No	13 (27.7%)	
	**Time of follow-up (months)**	81.36 ± 38.37

**Table 2:** Distribution of symptoms prompting medical evaluation at diagnosis.

Symptom Prompting Medical Evaluation at Diagnosis	Frequency
Fracture	30 (63.8%)
Skeletal deformities	10 (21.3%)
Antenatal diagnosis	2 (4.3%)
Blue sclerae	2 (4.3%)
Inguinal hernia	1 (2.1%)
Hypotonia	1 (2.1%)
Gait disturbances	1 (2.1%)

**Table 3:** Association between OI type and lower limb deformities.

OI Type	Limb Length Discrepancy *n* (%)	Coxa Vara *n* (%)
Type I (*n* = 20)	6 (30.0%)	6 (30.0%)
Type III (*n* = 14)	14 (100%)	12 (85.7%)
Type IV (*n* = 10)	6 (60.0%)	12 (85.7%)
Type V (*n* = 2)	2 (100%)	2 (100%)
Type XVII (*n* = 1)	0	0
Fisher’s exact test (*p*)	<0.001	=0.009

## Data Availability

Data are contained within the article.

## Abbreviations

The following abbreviations are used in this manuscript:

OI	Osteogenesis imperfecta
SD	Standard deviation
IQR	Interquartile range
